# An in‐house 45‐plex array for the detection of antimicrobial resistance genes in Gram‐positive bacteria

**DOI:** 10.1002/mbo3.1341

**Published:** 2022-12-25

**Authors:** Carole Kowalewicz, Michael Timmermans, David Fretin, Pierre Wattiau, Cécile Boland

**Affiliations:** ^1^ Veterinary Bacteriology, Sciensano Ixelles Belgium

**Keywords:** *Enterococcus*, *optrA*, *poxtA*, *Staphylococcus*, *vanA*, *vanL*

## Abstract

Identifying antimicrobial resistance (AMR) genes and determining their occurrence in Gram‐positive bacteria provide useful data to understand how resistance can be acquired and maintained in these bacteria. We describe an in‐house bead array targeting AMR genes of Gram‐positive bacteria and allowing their rapid detection all at once at a reduced cost. A total of 41 AMR probes were designed to target genes frequently associated with resistance to tetracycline, macrolides, lincosamides, streptogramins, pleuromutilins, phenicols, glycopeptides, aminoglycosides, diaminopyrimidines, oxazolidinones and particularly shared among *Enterococcus* and *Staphylococcus* spp. A collection of 124 enterococci and 62 staphylococci isolated from healthy livestock animals through the official Belgian AMR monitoring (2018–2020) was studied with this array from which a subsample was further investigated by whole‐genome sequencing. The array detected AMR genes associated with phenotypic resistance for 93.0% and 89.2% of the individual resistant phenotypes in enterococci and staphylococci, respectively. Although linezolid is not used in veterinary medicine, linezolid‐resistant isolates were detected. These were characterized by the presence of *optrA* and *poxtA*, providing cross‐resistance to other antibiotics. Rarer, vancomycin resistance was conferred by the *vanA* or by the *vanL* cluster. Numerous resistance genes circulating among *Enterococcus* and *Staphylococcus* spp. were detected by this array allowing rapid screening of a large strain collection at an affordable cost. Our data stress the importance of interpreting AMR with caution and the complementarity of both phenotyping and genotyping methods. This array is now available to assess other One‐Health AMR reservoirs.

## Graphical abstract

Here we describe an in‐house bead array targeting antimicrobial resistance (AMR) genes of Gram‐positive bacteria and allowing their rapid detection all at once at reduced costs. A total of 27/41 AMR probes targeting genes frequently associated with resistance tested positive on a collection of 124 enterococci and 62 staphylococci isolated from healthy livestock animals. The array detected AMR genes associated with phenotypic resistance for 93% and 89.2% of the individual resistant phenotypes in enterococci and staphylococci, respectively.

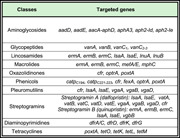

## 1 INTRODUCTION

Antimicrobial resistance (AMR) has become a major concern threatening public health (Nowakiewicz et al., 2019). For many decades, antibiotics are widely used in animal and human areas, leading to the worldwide resistance phenomenon. Indeed, bacteria have always been able to adapt by developing or acquiring mechanisms of resistance (Duval et al., 2019). In response to selective pressure and to survive antibiotic exposure, resistance occurs generally through the acquisition of genes located on mobile elements (Argudin et al., 2017; Strauss et al., 2015). Despite awareness associated with restrictive measures in animal production, the effects of the intensive use of drugs seem to persist lengthily, impacting numerous environments and ecological niches (Argudin et al., 2017; Nowakiewicz et al., 2019). Zoonotic, as well as commensal bacteria, became resistant providing them with a selective advantage (Argudin et al., 2017; Perreten et al., 2005) and sharing a potential risk of dissemination of AMR genes (De Jong et al., 2019; Nowakiewicz et al., 2019; Perreten et al., 2005).

Commensal bacteria, such as *Enterococcus* spp. are natural inhabitants of the gastrointestinal tract of healthy animals and humans. Enterococci can also be considered opportunistic pathogens when found related to human infections. They can persist in the environment and survive in different ecological niches such as soil, water, food items, and sewage (Nowakiewicz, 2019; Osman et al., 2019; Raza et al., 2018; Torres et al., 2018). Enterococci have emerged as one of the four most prevalent nosocomial human pathogens worldwide, especially due to their virulence, ability to form a biofilm, and intrinsic resistance (Leite‐Martins et al., 2015; Raza et al., 2018). Notably, *Enterococcus* spp. are intrinsically resistant to a number of antimicrobials including trimethoprim‐sulfamethoxazole, vancomycin (*Enterococcus gallinarum*, *Enterococcus casseliflavus*, and *Enterococcus flavescens*), streptogramins (*Enterococcus faecalis*) and exhibit low‐level resistance to ß‐lactams and aminoglycosides (Argudin et al., 2017; Torres et al., 2018; Zaheer et al., 2020). Since the 1980s, AMR enterococci have been the leading cause of hospital‐acquired bloodstream and urinary tract infections, mainly through biofilm formation on catheters and implanted medical devices (Argudin et al., 2017; De Jong et al., 2019; Mercuro et al., 2018; Raza et al., 2018; Torres et al., 2018; Zaheer et al., 2020). Particularly, the majority of enterococcal infections are caused by *E. faecalis* and *Enterococcus faecium*, the most common species encountered in the human gut (Argudin et al., 2017; Mercuro et al., 2018; Raza et al., 2018; Torres et al., 2018). *E. faecium* has become a prominent cause of nosocomial infections often characterized by high‐level resistance to multiple antibiotics (De Jong et al., 2019). Due to the high plasticity of enterococcal genomes, transfer and acquisition of AMR determinants in enterococci and other Gram‐positive bacteria are then facilitated (Leite‐Martins et al., 2015; Nowakiewicz et al., 2019).

*Staphylococcus* spp. are commensal bacteria widely found on the skin or mucosal surfaces of animals and humans (Alharbi, 2019; Bortolaia et al., 2016; Craft et al., 2019; Dastgheyb & Otto, 2015; Wendlandt et al., 2013). Despite preventing colonization by pathogenic bacteria (Alharbi, 2019), staphylococci are opportunistic pathogens often responsible for chronic and severe nosocomial infections (Bortolaia et al., 2016; Dastgheyb & Otto, 2015). Specifically, *Staphylococcus aureus* is one of the most pathogenic bacteria associated with human and animal diseases causing persisting skin and soft tissue infections (SSTIs), infectious endocarditis, septic arthritis, and osteomyelitis (Alharbi, 2019; Craft et al., 2019; Dastgheyb & Otto, 2015). The colonization of skin or mucosal surfaces by methicillin‐resistant *S. aureus* (MRSA) and its dissemination in healthcare settings represents a global health issue (Holmes et al., 2015; Watkins et al., 2019) especially due to its capacity to acquire new AMR and to spread rapidly (Alharbi, 2019; Holmes et al., 2015). Indeed, methicillin resistance was first observed among clinical isolates before its rapid spread to the community (Turner et al., 2019). *Staphylococcus* spp. may exchange resistance determinants to numerous other bacteria in the same animal or human host, or between hosts by direct contact or through excretions such as sneezing, coughing, or licking (Wendlandt et al., 2013). Even if MRSA‐associated infection rates have declined, these human infections are still problematic since they are characterized by broadening AMR and high rates of hospitalization and mortality (Purrello et al., 2016).

Over time, AMR has widely spread, consequently leaving healthcare personnel with last‐line antibiotics (i.e., vancomycin, linezolid, and daptomycin) as the preferred means, and sometimes the only options to treat multidrug‐ resistant (MDR) infections (Raza et al., 2018; Sadowy, 2018; Torres et al., 2018; Zaheer et al., 2020). Resistance to last‐resort drugs was already observed as well (Azhar et al., 2017; Doern et al., 2016; Holmes et al., 2015; Purrello et al., 2016) and among various bacteria, mainly reported in clinical settings (Argudin et al., 2017; Zaheer et al., 2020). Specifically, due to the alarming worldwide emergence (Osman et al., 2019; Raza et al., 2018), vancomycin‐resistant enterococci (VRE) was ranked as a pathogen of high priority by the World Health Organization (WHO) (Wist et al., 2020). Due to its ability to confer high levels of vancomycin‐resistance, *vanA* is the most reported gene among clinical VRE (Azhar et al., 2017; Turner et al., 2019; Watkins et al., 2019) and widely spread to other co‐infecting bacteria such as *S. aureus*. Despite restricted use, resistance to linezolid has been reported in various species, strains, and settings (Sadowy, 2018). Determinants associated with linezolid resistance such as the highly mobilizable *cfr* coding for a ribosomal methyltransferase conferring cross‐resistance to phenicols, lincosamides, oxazolidinones, pleuromutilins, and streptogramin A or the oxazolidinone‐phenicol transferable resistance gene *optrA* have been identified in staphylococci, enterococci and other Gram‐positive species isolated from both humans and animals (Azhar et al., 2017; Doern et al., 2016; Sadowy, 2018; Timmermans et al., 2022a; Torres et al., 2018). The most recently described *poxtA* mediating decreased susceptibility to oxazolidinones, phenicols, and tetracyclines has also been detected in different Gram‐positive bacteria of animal and human origin (Brenciani et al., 2018; Ruiz‐Ripa et al., 2020; Sadowy, 2018; Timmermans et al., 2022a).

In summary, AMR is not restricted to a particular species or host. It became essential to consider all bacteria as a potential pool of resistance determinants possibly transferable to other pathogenic or commensal bacteria both in animals and humans (Argudin et al., 2017; De Jong et al., 2019; Osman et al., 2019; Perreten et al., 2005; Torres et al., 2018; Zaheer et al., 2020). Therefore, it is important to identify AMR determinants spreading in humans and animals (Perreten et al., 2005; Strauss et al., 2015) and to consider them as a One Health AMR pool. The complexity of AMR and particularly the cross‐resistance phenomenon, that is resistance to multiple distinct antimicrobial classes conferred by a single molecular mechanism, requires monitoring all putative main sources of AMR at the genetic level.

We describe an in‐house developed array targeting major AMR genes of Gram‐positive bacteria and allowing their rapid and efficient detection all at once at reduced costs. In this study, we aimed to target AMR genes commonly found and particularly shared among *Enterococcus* and *Staphylococcus* spp. The diversity of probes per antimicrobial class reflects phenotypes observed during several years of field monitoring and pointed out the most critical (e.g., glycopeptides) or emergent ones (e.g., linezolid) to investigate. The presence of these AMR genes was studied in a collection of 124 enterococci and 62 staphylococci isolated from healthy livestock animals through the official Belgian AMR monitoring during the period 2018–2020, to decipher the genetic nature of this pool of AMR and thereby provide useful data to understand how resistance can be acquired and maintained in these bacteria.

## 2 MATERIALS AND METHODS

### 2.1 Isolate collection

All *Enterococcus* spp. and MRSA isolates included in this study were collected during the voluntary Belgian AMR monitoring programs of 2019 and 2020 and 2018 and 2019, respectively, from healthy food‐producing animals by the Federal Agency for the Safety of the Food Chain (FASFC, 2020, http://www.favv.be/productionanimale/antibioresistance/resultats/#sciensano). *Enterococcus* spp. were isolated from fecal samples of pigs (*N* = 15 *E. faecalis* and 8 *E. faecium*), veal calves (*N* = 22 *E. faecalis* and 16 *E. faecium*), turkeys (*N* = 4 *E. faecalis* and 7 *E. faecium*) and broilers (*N* = 9 *E. faecalis* and 30 *E. faecium*) collected at a slaughterhouse and of chicken breeding hens (*N* = 4 *E. faecalis* and 6 *E. faecium*) and laying hens (*N* = 3 *E. faecalis*) collected at farm. MRSA have been isolated from nasal swab samples of sows (*N* = 26), fattening pigs (*N* = 25), or calves (*N* = 11) collected at the farm.

The selection of isolates was based on phenotypic resistance profiles determined by broth microdilution (BMD) assay carried out during the annual monitoring by Sciensano and interpreted according to European Food Safety Authority (EFSA) guidelines related to the European decision (2013/652/EU). Among selected staphylococci and enterococci isolates, all were multi‐resistant and/or displayed phenotypic resistance to critical antibiotics such as vancomycin or linezolid. All selected MRSA were resistant to at least six different antimicrobial classes, including β‐lactams (i.e., penicillin and cefoxitin). Resistance to antimicrobials included in both *Enterococcus* spp. and *S. aureus* testing panels (EUVENC and EUST plates, SensititreTM; Thermo Fisher Scientific) was targeted by this array (see Table 1) and directed isolate selection. Most targeted resistance genes are shared by both enterococci and staphylococci. Control isolates (*N* = 25) originated from the European Reference Laboratory for Antimicrobial Resistance (EURL‐AR), from Sciensano internal collection, or other sources as listed in Table A1.

**Table 1:** List of the AMR genes targeted by the array and their related antimicrobials and antimicrobial classes monitored by broth microdilution in *Enterococcus* spp. and/or *Staphylococcus* spp.

Classes	Antimicrobials	Targeted genes
Aminoglycosides	Kanamycin^a^_1_ (KAN)	*aadD* ^*a*^, *aadE* ^*c*^, *aacA‐aphD* ^*ab*^, *aphA3* ^*a*^, *aph2‐Id* ^*ab*^, *aph2‐Ie* ^*bc*^
	Gentamicin^b^ (GEN)	
	Streptomycin^c^_1_ (STR)	
Glycopeptides	Vancomycin (VAN)	*vanA*, *vanB*, *vanC*_*1*_, *vanC*_*2‐3*_
Lincosamides	Clindamycin_1_ (CLN)	*ermA*, *ermB*, *ermC*, *lsaA*, *lsaE*, *lnuA*, *lnuB*
Macrolides	Erythromycin (ERY)	*ermA*, *ermB*, *ermC*, *mefA/E*, *mphC*
Oxazolidinones	Linezolid (LZD)	*cfr*, *optrA*, *poxtA*
Phenicols	Chloramphenicol (CHL)	*catp*_*C194*_, *catp*_*C221‐223*_, *cfr*, *fexA*, *optrA*, *poxtA*
Pleuromutilins	Tiamulin_1_ (TIA)	*cfr*, *lsaA*, *lsaE*, *vgaA*, *vgaB*, *vgaD*,
Streptogramins	Quinupristin (Group B—streptogramin)/dalfopristin (Group A—streptogramin) (Synercid [SYN])	*Streptogramin A (dalfopristin): lsaA*, *lsaE*, *vatA*, *vatB*, *vatC*, *vatD*, *vatE*, *vgaA*, *vgaB*, *vgaD*, *cfr* *Streptogramin B (quinupristin): ermA*, *ermB*, *ermC*, *lsaA*, *lsaE*, *vgbB*
Diaminopyrimidines	Trimethoprim_1_ (TMP)	*dfrA/C*, *dfrD*, *dfrK*, *dfrG*
Tetracyclines	Tetracycline (TET)	*poxtA*, *tetO*, *tetK*, *tetL*, *tetM*

_1_Antimicrobials monitored by broth microdilution in *Staphylococcus* spp. only.

### 2.2 Antimicrobial susceptibility testing (AST)

Antimicrobial minimum inhibitory concentrations (MICs) were determined by BMD with EUVENC (for enterococci) and EUST (for staphylococci) multiwell plates (Sensititre™; Thermo Fisher Scientific) and interpreted according to the EFSA guidelines as detailed in ad hoc reports (Federal Agency for the Safety of the Food Chain, 2020, http://www.favv.be/productionanimale/antibioresistance/resultats/#sciensano) and based on epidemiological cut‐off values (ECOFFs). Antimicrobial susceptibility testing (AST) for clindamycin, kanamycin, streptomycin, tiamulin, and trimethoprim was performed in *S. aureus* only.

### 2.3 AMR genes detection with the in‐house array

The molecular method developed for the detection of AMR genes is a multiplex assay based on a Ligase Chain Reaction (LCR) of Padlock‐shaped Probes (PLPs) followed by hybridization of LCR products with MagPlex‐TAG™ microspheres coated with unique 24‐nt long capture tags and detection with a Luminex® 200™ instrument (Luminexxas).

#### 2.3.1 PLPs probes design

Probes were designed as described previously (Boland et al., 2018; Timmermans et al., 2022b; Wattiau et al., 2011). The sequences of the probes developed in this study and used in the LCR assay and the corresponding AMR genes targeted by the array are listed in Table 2. Their design was based on published DNA sequences of AMR genes from the NCBI nucleotide database (https://www.ncbi.nlm.nih.gov/nuccore). Several sequences of the same AMR gene from both *Enterococcus* and *Staphylococcus* spp. were aligned with Bionumerics 6.6 (bioMérieux SA) and target‐specific sequences were selected within the most conserved regions. In addition to these specific sequences corresponding to the two extremities of the probes, the so‐called 5'‐arms and 3’‐arms, probes encompassed: sequences of the universal primers “reverse” (cUR) and “forward” (UF; see Table 2) as well as an anti‐TAG sequence matching a given TAG sequence of the MagPlex‐TAG™ microspheres (Luminex). Probes are schematically represented in linear form as follows: **5'arm – cUR – AA – UF – anti‐TAG – 3'arm** where “AA” is a di‐deoxyadenosine linker.

**Table 2:** Probes used for the LCR assay and IDs of the corresponding Luminex® MagPlex‐TAG™ microspheres

**Probes**	**MagPlex‐TAG™ microspheres**	**Final conc. pM** b	**Sequence**
*aacA‐aphD*	MTAG‐A022	400	**TTTGCCAGAACATGAATTACACGAGGG**‐cUR‐AA‐UF‐CAAACAAACATTCAAATATCAATC **ACCAAAAATCTGGTTTTAGAATTATTGAAGA**
*aadD*	MTAG‐A047	200	**GTCTGACGACCAAGAGAGCCATAAACA**‐cUR‐AA‐UF‐TCTCTTTAAACACATTCAACAATA **ATATCCGAATAGGGCCCATCA**
*aadE*	MTAG‐A056	200	**TCATGTAAGAAAGCCAAGCGCAAGG**‐cUR‐AA‐UF‐CTTAAACTCTACTTACTTCTAATT **GGGACATAGTTCCGACTGATATAGATTA**
*aph2‐Id/aph2‐Ie*	MTAG‐A027	200	**TGCAGCTATTTCTGATCCCGACAATG**‐cUR‐AA‐UF‐TAACTTACACTTAACTATCATCTT **CATTTGTGGAATAATCGATTTTGGAGA**
*aphA3*	MTAG‐A028	200	**TGGCTGGAAGGAAAGCTGCC**‐cUR‐AA‐UF‐CACTTAATTCATTCTAAATCTATCA **CGGGAAAAGGACATGATGCTA**
*cat*p_C194_	MTAG‐A021	400	**ACTGGTTACAATAGCGACGGAGAG**‐cUR‐AA‐UF‐TCAAACTCTCAATTCTTACTTAAT **GGTGATAAACTCAAATACAGCTTTTAGA**
*cat*p_C221_,*/cat*p_C223_	MTAG‐A026	200	**CCATACCGATTTCAATGATTCCTTGGATTG**‐cUR‐AA‐UF‐TACATTCAACACTCTTAAATCAAA **CCTAAAAAACCGATACCTGAAAACA**
*cfr*	MTAG‐A020	200	**TGCAAACGAAGTTGTTAGCCTTCTTAAAAG**‐cUR‐AA‐UF‐CTTTCTCATACTTTCAACTAATTT **GGTGTAAATGATTCTCTTGAGCA**
*dfrA/dfrC*	MTAG‐A034	200	**ACCAAGCTTCATTTCACCATGAAGGG**‐cUR‐AA‐UF‐ACTTATTTCTTCACTACTATATCA **AGACGTAACGTCGTACTCACTA**
*dfrD*	MTAG‐A035	200	**ATCGGAAGGGCTTTACCTGACAGAA**‐cUR‐AA‐UF‐CATCTTCATATCAATTCTCTTATT **AATCATATTAGGTAGAAAGAACCTTCAATCA**
*dfrG*	MTAG‐A044	400	**GAATGACATTCCTTGGAGGATTCCCAA**‐cUR‐AA‐UF‐TCATCACTTTCTTTACTTTACATT **GATAAGAATAGAGTGATTGGCAAAGA**
*dfrK*	MTAG‐A042	200	**TCTTGAATCAATCGGAAGAGCA**‐cUR‐AA‐UF‐CACTACACATTTATCATAACAAAT **AGGGATATCCAATTATATTAGGAAGGAAGAA**
*ermA*	MTAG‐A046	800	**TTTGCATGCTTCAAAGCCTGTCG**‐cUR‐AA‐UF‐TTAAACAATCTACTATTCAATCAC **TCCTTCGATAGTTTATTAATATTAGTGACA**
*ermB*	MTAG‐A061	200	**AGTCTCGATTCAGCAATTGCTTAAGCT**‐cUR‐AA‐UF‐AATCTCTACAATTTCTCTCTAATA **GTTGCTCTTGCACACTCA**
*ermC* a	MTAG‐A014	200	**TTAGTACAGAGGTGTAATTTCGTAACTGCC**‐cUR‐AA‐UF‐AATTTCTTCTCTTTCTTTCACAAT **GAAAAGGSCATTTTACCCTTGAA**
*fexA*	MTAG‐A029	200	**TGATATTGTTGCCAGGTGGTGTGG**‐cUR‐AA‐UF‐TACTACTTCTATAACTCACTTAAA **CTTCTGGACAGGCTGGAA**
*Gram*+	MTAG‐A052	200	**CCTTCCTCCGGTTTGTCACC**‐cUR‐AA‐UF‐TTCTTCATTAACTTCTAATCTTAC **ATGATTTGACGTCATCCCCA**
*lnuA*	MTAG‐A074	800	**GATATAGATTTTGACGCTCAACACACTCAA**‐cUR‐AA‐UF‐ACACTCATTTAACACTATTTCATT **AAACAACAAAGAGAACACAGAGATATA**
*lnuB*	MTAG‐A075	400	**TGGATCGTTTACCAAAGGAGAAGGTGAC**‐cUR‐AA‐UF‐CATAAATCTTCTCATTCTAACAAAA **TGAACGAATTACAGCTTGTATGATGTA**
*lsaA*	MTAG‐A036	400	**TTGACGGTGGAAGAGCTTCGTC**‐cUR‐AA‐UF‐ATTAAACAACTCTTAACTACACAA **GCCAACGCATCACAAAACATTA**
*lsaE*	MTAG‐A012	400	**TGTTGGACATAAGGCTGCAAAAGCG**‐cUR‐AA‐UF‐CATAATCAATTTCAACTTTCTACT **CTGGTTCAAAACTGGATAAGGGTTA**
*mefA/mefE* a	MTAG‐A045	200	**GCAATTGTACGTATWCCTAAGCTGGGT**‐cUR‐AA‐UF‐TACACAATATTCATCATAACTAAC **GCTGTGATTGCATCTATTACGGTA**
*mphC*	MTAG‐A063	200	**TCGCTGAGTTTGCTATGGAATCAGGAG**‐cUR‐AA‐UF‐CTAAATCACATACTTAACAACAAA **ACTCAATGCAGTATTCCCAATGTTTA**
*optrA*	MTAG‐A018	400	**GAAGGAGAAGGTTAAGAAGGAGAAACG**‐cUR‐AA‐UF‐ACACTTATCTTTCAATTCAATTAC **GCGATCGTAACTCCATTGA**
*poxtA*	MTAG‐A055	200	**CCAGTGGAAATGCCCGTATTGGTTAT**‐cUR‐AA‐UF‐ACATCAAATTCTTTCAATATCTTC **CTTGAACTTGATAATGGTTCACTGA**
*sodA‐fm* **C**	MTAG‐A054	800	**AGACAGCTGTACGTAACAATGGTGG**‐cUR‐AA‐UF‐TTAATACAATTCTCTCTTTCTCTA **GGACGCTATTCCAACAGATATCA**
*sodA‐fs* **C**	MTAG‐A057	800	**TGGTGGCGGTCACGC**‐cUR‐AA‐UF‐ACTTACAATAACTACTAATACTCT **CGTACAGCCGTTCGTAACAA**
*staph* **C**	MTAG‐A053	200	**TGCTACGGTGAATACGTTCCCG**‐cUR‐AA‐UF‐TTAACAACTTATACAAACACAAAC **TCGCTAGTAATCGTAGATCAGCA**
*tetK*	MTAG‐A066	200	**TTCCCTTCACTGATTATGGTGGTTGTAGC**‐cUR‐AA‐UF‐TCTTACTAATTTCAATACTCTTAC **AGGAGTAGGATCTGCTGCA**
*tetL*	MTAG‐A067	400	**GCTAAGTACTGCCGAAATCGGAAGTG**‐cUR‐AA‐UF‐ATCTCAATTACAATAACACACAAA **GTTCCTTATATGATGAAAGATGTTCACCA**
*tetM*	MTAG‐A072	400	**AAGGTACAACGAGGACGGATAATACGC**‐cUR‐AA‐UF‐CTATCATTTATCTCTTTCTCAATT **CAGAATTAGGAAGCGTGGACA**
*tetO*	MTAG‐A065	200	**GACAGCAGTGACATCTTTTCAGTGGG**‐cUR‐AA‐UF‐TACTTAAACATACAAACTTACTCA **GTCAAAGGGGAATCACTATCCA**
*vanA*	MTAG‐A013	200	**AGTCAATACTCTGCCCGGT**‐cUR‐AA‐UF‐CAAATACATAATCTTACATTCACT **GCCGCATTGTACTGAACGA**
*vanB*	MTAG‐A033	200	**ATGCGGGCATCGCCG**‐cUR‐AA‐UF‐ACTACTTATTCTCAAACTCTAATA **TCACTGGCCTACATTCTTACAAAAA**
*vanC*_*1*_	MTAG‐A030	200	**TGGGAATCGCTAGTGCTCCCAC**‐cUR‐AA‐UF‐CTTAACATTTAACTTCTATAACAC **TCTTGCATCAACTTGCTGATACCA**
*vanC*_*2*_ */vanC*_*3*_ a	MTAG‐A051	200	**TCGAAGCACTCCAATCATCTCCC**‐cUR‐AA‐UF‐CAATTTACATTTCACTTTCTTATC **TTAGCYTCAGCAACTAGCGCAA**
*vatA*	MTAG‐A073	200	**TTGCTGCAGAAGCTGTTGTCACAAAG**‐cUR‐AA‐UF‐CTTTATCAAATTCTAATTCTCAAC **GGGACGGGGCAATCA**
*vatB*	MTAG‐A039	200	**ATTCAAATAGGAGATGGAGCAATTGTTGCT**‐cUR‐AA‐UF‐ACAAATATCTAACTACTATCACAA **AGAATGTTACTGTTATGCCAGGA**
*vatC*	MTAG‐A078	200	**ATGGTTGGGAGAAGCATACCCCTA**‐cUR‐AA‐UF‐TTTACAAATCTAATCACACTATAC **CAACATTTCCATTCAATCTTTTCGGAA**
*vatD*	MTAG‐A077	200	**GCGCCATACATGTTAGCTGGAGGAA**‐cUR‐AA‐UF‐AATAACAACTCACTATATCATAAC **GCTGCTAATTCTGTTGTTGTAAAAGATATA**
*vatE*	MTAG‐A076	200	**CCGATTTTGAGAAACACGTTACCCATCAC**‐cUR‐AA‐UF‐TCTCATCTATCATACTAATTCTTT **CTATTATGATGACCCAGTAAATCCCA**
*vgaA*	MTAG‐A025	200	**CTCAGTCGATTGAGTATTGAACCTTCGGAA**‐cUR‐AA‐UF‐CTTTCTTAATACATTACAACATAC **ACTTTTACTTGAGACAAAAATTACAGAAGTA**
*vgaB*	MTAG‐A038	200	**ATAAGGCGCAAGGAATGATTAAGCCC**‐cUR‐AA‐UF‐ATTCAATACTATCTAACACTTACT **GGAGCAAGCTATAAAGCTAAAAGAGA**
*vgaD* c	MTAG‐A043	200	**TTGTTTGCCGACGAACCAACTACAAAC**‐cUR‐AA‐UF‐AACTTTCTCTCTCTATTCTTATTT **CATTAGCGATGAAGGCGGAAATA**
*vgbB*	MTAG‐A048	200	**TCACTAGTGGTAACGATGGTGCAC**‐cUR‐AA‐UF‐AATCAACACACAATAACATTCATA **AGCGGCTCCAGTGGGTA**
UF			GTAGACTGCGTACCAATTC
UR			GACGATGAGTCCTGAGTAA
cUR			TTACTCAGGACTCATCGTC

*Note*: Nucleotide sequence of the probe (from 5' to 3'). Bold characters highlight the sequence targeted on the template DNA, underlined characters indicate the sequence complementary to the Luminex® MagPlex‐TAG™microspheres with the bead ID indicated in the second column; UF, UR, and cUR, nucleotide sequence of the PCR amplification primers Universal Forward, Reverse and complementary UR, respectively (Wattiau et al., 2011; Boland et al., 2018); normal characters indicate nucleotides added to reach a final set of probes with evenly distributed sizes ranging from 99 to 125 nucleotides. For the design of each probe, the most conserved region of the targeted gene, obtained from alignments in Bionumerics© of sequences available in GenBank, was chosen as the target sequence. Three control probes (indicated with the letter “C”) were designed for the identification of each bacterial species, *Enterococcus faecalis*, *Enterococcus faecium*, and *Staphylococcus aureus*.

^a^ Nucleotide sequence including a wobble.

^b^ Final concentration in the first step of the Ligase Chain Reaction (LCR) assay.

^c^ No control was available to validate this PLP.

#### 2.3.2 DNA preparation

The DNA samples were prepared from bacteria grown on Columbia Sheep Blood agar plates (Oxoid) with the QIAGEN DNeasy blood and tissue kit (Qiagen) according to the manufacturer's instructions for Gram‐positive bacteria. Both DNA purity and concentration were assessed using the Nanodrop 1000 (Isogen Life Science) before storage at −20°C.

#### 2.3.3 LCR assay

The LCR assay was conducted in three successive steps according to the procedure of Boland et al. (2018) with minor modifications. The first step (ligation) was conducted in a 10 µL mixture containing 1 µL of *Pfu* DNA ligase buffer 10X (#600191‐52; Agilent), 2 U of *Pfu* DNA ligase (#600191‐51; Agilent), a specific final concentration of each PLP detailed in Table 2 and 1 µL of DNA (≥10 ng μL^−1^) extracted as described above. The thermoCycler conditions were as follows: denaturation at 95°C for 3 min followed by 25 cycles of 30 s at 95°C and 5 min at 65°C and 2 min at 98°C for final denaturation. The second step (exonuclease treatment) consisted of the addition of 15 µl of exonuclease mixture containing 67 mM glycine‐ KOH, pH 9.4, 2.5 mM MgCl_2_, 50 μg mL^−1^ bovine serum albumin (BSA) (#B0262S; New England BioLabs) and 0.0015 U λ exonuclease (#M262S; New England BioLabs) to the step 1 LCR products. The resulting 25‐µL sample was incubated at 37°C for 45 min followed by a 10 min inactivation at 95°C. As the third step, PCR amplification was performed by adding a mix of 50 µL of 2x Absolute qPCR mixture (Thermo Scientific) supplemented with 2 µL of universal reverse UR primer concentrated at 2.5 µM and 2 µL 5'Cy3‐labeled universal forward UF primer concentrated at 20 µM to each sample. After 10 min denaturation at 95°C, 30 cycles of 45 s at 95°C, 45 s at 55°C and 1 min at 72°C were conducted and followed by 15 min of final elongation at 72°C and 2 min of denaturation at 98°C. LCR products were stored at −20°C until hybridization and reading on a bead array platform.

#### 2.3.4 Hybridization and detection of the LCR products on a bead‐array platform

A bead mix was prepared in TE buffer pH 8 to obtain a final concentration of 1 x10^5^ beads per mL per region of each MagPlex‐TAG™ microsphere listed in Table 2 and an additional bead unrelated to the probes and used as negative hybridization control. The bead mix was stored at 4°C and protected from light.

Before hybridization, the bead mix was pelleted on a magnet and suspended in 2× hybridization buffer (0.4 M NaCl, 0.2 M Tris, 0.16% Triton X‐100, pH 8.0) at a concentration of 50 beads of each type per µL. The hybridization step consisted in mixing 25 µL of the bead mix and 25 µL of the final LCR product followed by 90 s of denaturation at 96°C and 30 min of hybridization at 37°C.

Right after hybridization, three washes were performed by pelleting the beads on a magnetic bead separation system (V&P Scientific) for 1 min, removing the supernatant by forceful inversion, and suspending the beads in 75 µl 1× hybridization buffer (0.2 M NaCl, 0.1 M Tris, 0.08 Triton X‐100, pH 8.0). After the final wash, the plate was incubated at 37°C for 15 min in the Luminex® 200™ instrument.

Bead‐array analysis was performed on 50 µL of the final solution at 37°C and Median Fluorescence Intensity (MFI) was measured on at least 100 beads of each bead type.

#### 2.3.5 Data analysis

The fluorescence signal of the Gram‐positive control probe “Gram+” was used as an internal standard to normalize MFI signals observed for each sample according to the formula: (MFI probe/MFI Gram+) × 100. The results expressed in normalized MFI (nMFI) were evaluated against cut‐offs. Cut‐offs were determined after plotting experimental nMFI values obtained by testing isolates and control strains and included a twofold ratio between positive and negative nMFI. The probes of the array were validated with reference strains used as positive controls, except for the *vgaD* probe for which no reference strain was available (see Table A1). The control probes *soda_fs* and *soda_fm* are based on the housekeeping *sodA* gene of *E. faecalis* and *E*. *faecium*, respectively*;* the *staph* control probe is based on the 16S rRNA gene of *S. aureus*. Control probes were validated on *E. faecalis* ATCC 29212, *E. faecium* 2013/16227 field strain, and *S. aureus* ATCC 29213 reference strains. These three control strains together with the negative control *Escherichia coli* ATCC 25922 were used in each experiment to validate the LCR assay.

### 2.4 Next‐generation sequencing analysis

A total of 31 isolates (16 *E. faecalis*, 14 *E. faecium*, and 1 MRSA) were analyzed by whole‐genome sequencing to further investigate linezolid and vancomycin‐resistant isolates and a few isolates resistant to five or more antimicrobials. Isolate selection was based on the resistance phenotypes, irrespective of the bacterial species, that is all isolates resistant to linezolid (11 *E. faecalis* and 11 *E. faecium*) and vancomycin (*n* = 2 *E. faecalis*). The remaining isolates (3 *E. faecalis*, 3 *E. faecium*, and 1 MRSA) were selected because of their MDR phenotype (resistant to at least five different antimicrobials). Genomic DNA was extracted as detailed in “DNA preparation.” Libraries were prepared using Nextera XT DNA library preparation kit (Illumina) according to the manufacturer's instructions and underwent Illumina sequencing using the MiSeq V3 chemistry (Illumina) for the production of two ×250 bp paired‐end reads. Raw sequenced reads were trimmed with Trimmomatic v0.38 using default settings and de novo assembled with SPAdes v 3.13.0 using default settings (Bankevich et al., 2012). Identification of AMR genes was assessed with ResFinder 4.1 using default settings (Bortolaia et al., 2020).

## 3 RESULTS

In this study, a bead array based on AMR genes was developed and validated on reference strains (see Table A1) to characterize the genetic nature of resistance expressed by Gram‐positive bacteria. This array was used to detect AMR genes in a selection of enterococci and staphylococci isolated from livestock animals in Belgium. A total of 186 Gram‐positive bacteria including 57 *E. faecalis*, 67 *E. faecium*, and 62 MRSA were screened for the presence of the AMR genes detailed in Table 1. Altogether, 27 out of the 41 AMR probes tested positive on the 186 selected animal isolates (see Tables 3 and 4). In most cases, resistance profiles correlated with markers detected with the array. Results of this AMR gene screening are presented and discussed hereafter per antimicrobial class, followed by whole genome sequencing (WGS) analysis results conducted to further investigate linezolid and vancomycin‐resistant isolates and a few isolates resistant to five or more antimicrobials.

**Table 3:** The number of isolates carrying the resistance genes detected by the array per species in *Enterococcus faecalis*, *Enterococcus faecium* and *Staphylococcus aureus* among resistant isolates and classified per antimicrobial

**AMR genes**	R‐*E. faecalis*	R‐*E. faecium*	R‐*S. aureus*	**AMR genes**	R‐*E. faecalis*	R‐*E. faecium*	R‐*S. aureus*
**Chloramphenicol (CHL)**	33	13	12	**Quinupristin/dalfopristin (SYN)** continued	57	53	37
*catp*_C194_	0 (0.0%)	2 (15.4%)	0 (0.0%)	*lsaE*	20 (35.1%)	37 (69.8%)	29 (78.4%)
*catp*_C221/223_	4 (12.1%)	1 (7.7%)	0 (0.0%)	*vatA*	0 (0.0%)	0 (0.0%)	0 (0.0%)
*cfr*	0 (0.0%)	0 (0.0%)	1 (8.3%)	*vatB*	0 (0.0%)	0 (0.0%)	0 (0.0%)
*fexA*	17 (51.5%)	5 (38.5%)	9 (75.0%)	*vatC*	0 (0.0%)	0 (0.0%)	0 (0.0%)
*optrA*	18 (54.4%)	8 (61.5%)	0 (0.0%)	*vatD*	1 (1.8%)	0 (0.0%)	0 (0.0%)
*poxtA*	1 (3.0%)	7 (53.8%)	0 (0.0%)	*vatE*	0 (0.0%)	0 (0.0%)	0 (0.0%)
**Clindamycin (CLN)**	NT	NT	57	*vgaA*	0 (0.0%)	0 (0.0%)	0 (0.0%)
*ermA*	–	–	3 (5.3%)	*vgaB*	0 (0.0%)	0 (0.0%)	0 (0.0%)
*ermB*	–	–	15 (26.3%)	*vgaD*	0 (0.0%)	0 (0.0%)	0 (0.0%)
*ermC*	–	–	13 (22.8%)	*vgbB*	0 (0.0%)	0 (0.0%)	0 (0.0%)
*lsaA*	–	–	0 (0.0%)				
*lsaE*	–	–	29 (50.9%)	**Streptomycin (STR)**	NT	NT	11
*lnuA*	–	–	1 (1.8%)	*aadE*	–	–	4 (36.4%)
*lnuB*	–	–	26 (45.6%)	*aph2‐Id/Ie*	–	–	0 (0.0%)
**Erythromycin (ERY)**	52	54	36	**Tetracycline (TET)**	53	65	61
*ermA*	12 (23.1%)	25 (46.3%)	3 (8.3%)	*poxtA*	1 (1.9%)	12 (18.5%)	0 (0.0%)
*ermB*	49 (94.2%)	49 (90.7%)	15 (41.7%)	*tetK*	0 (0.0%)	0 (0.0%)	42 (68.9%)
*ermC*	0 (0.0%)	0 (0.0%)	13 (36.1%)	*tetL*	41 (77.4%)	51 (78.5%)	23 (37.7%)
*mefA/E*	0 (0.0%)	1 (1.9%)	0 (0.0%)	*tetM*	47 (88.7%)	65 (100.0%)	59 (96.7%)
*mphC*	0 (0.0%)	0 (0.0%)	0 (0.0%)	*tetO*	9 (17.0%)	0 (0.0%)	0 (0.0%)
**Gentamicin (GEN)**	25	12	19	**Tiamulin (TIA)**	NT	NT	35
*aacA‐aphD*	25 (100.0%)	12 (100.0%)	17 (89.5%)	*cfr*			1 (2.9%)
*aph2‐Id/Ie*	0 (0.0%)	0 (0.0%)	0 (0.0%)	*lsaA*	–	–	0 (0.0%)
**Kanamycin (KAN)**	NT	NT	19	*lsaE*	–	–	29 (82.9%)
*aacA‐aphD*	–	–	17 (89.5%)	*vgaA*	–	–	0 (0.0%)
*aadD*	–	–	13 (68.4%)	*vgaB*	–	–	0 (0.0%)
*aph2‐Id/Ie*	–	–	0 (0.0%)	*vgaD*	–	–	0 (0.0%)
*aphA3*	–	–	0 (0.0%)	**Trimethoprim (TMP)**	NT	NT	55
**Linezolid (LZD)**	11	11	0	*dfrA/C*	–	–	1 (1.8%)
*cfr*	0 (0.0%)	0 (0.0%)	0 (0.0%)	*dfrD*	–	–	0 (0.0%)
*optrA*	11 (100.0%)	9 (81.8%)	0 (0.0%)	*dfrG*	–	–	25 (45.5%)
*poxtA*	0 (0.0%)	8 (72.7%)	0 (0.0%)	*dfrK*	–	–	23 (41.8%)
**Quinupristin/dalfopristin (SYN)**	57	53	37	**Vancomycin (VAN)**	2	0	0
*ermA*	12 (21.1%)	20 (37.7%)	3 (8.1%)	*vanA*	1 (50.0%)	0 (0.0%)	0 (0.0%)
*ermB*	49 (86.0%)	35 (66.0%)	6 (16.2%)	*vanB*	0 (0.0%)	0 (0.0%)	0 (0.0%)
*ermC*	0 (0.0%)	0 (0.0%)	7 (18.9%)	*vanC*_*1*_	0 (0.0%)	0 (0.0%)	0 (0.0%)
*lsaA*	57 (100.0%)	0 (0.0%)	0 (0.0%)	*vanC*_*2‐3*_	0 (0.0%)	0 (0.0%)	0 (0.0%)

*Note*: For each antimicrobial, the numbers in the first line correspond to the number of isolates that were phenotypically resistant to this antimicrobial and assessed with the array. In front of each gene, the numbers in each column indicate the number of isolates in which this gene was detected with the array and the corresponding percentage of isolates carrying this gene among the number of isolates indicated in the top line for each antimicrobial. A dash indicates that the presence of the gene in the corresponding bacterial species was not assessed because the phenotypic resistance profile was not available.

Abbreviation: NT, not tested.

**Table 4:** The number of isolates carrying the resistance genes detected per species among *Enterococcus faecalis* and *Enterococcus faecium* isolates with undetermined phenotypes and classified per antimicrobial

AMR genes	*E. faecalis* (*n* = 57)	*E. faecium* (*n* = 67)
**Clindamycin (CLN)**	**ND**	**ND**
*lnuA*	0	0
*lnuB*	19	38
**Kanamycin (KAN)**	**ND**	**ND**
*aacA‐aphD*	26	13
*aadD*	1	2
*aph2‐Id/Ie*	0	0
*aphA3*	35	24
**Streptomycin (STR)**	**ND**	**ND**
*aadE*	33	42
*aph2‐Id/Ie*	0	0
**Trimethoprim (TMP)**	**ND**	**ND**
*dfrA/C*	0	0
*dfrD*	1	0
*dfrG*	35	26
*dfrK*	0	2

Abbreviation: ND, not determined.

### 3.1 Streptogramins resistance

Quinupristin/dalfopristin (Q/D; a streptogramins B and A combination) resistance is correlated with the presence of *lsa* (for Lincosamides Streptogramins A resistance) genes family. Particularly, intrinsic in *E. faecalis*, this resistance is conferred by expression of the *lsaA* gene (Frye & Jackson, 2013; Hollenbeck & Rice, 2012; Singh et al., 2002; Torres et al., 2018), found in all *E. faecalis* (*n* = 57/57) of this study. Concordant with this specificity, *lsaA* was not detected in *E. faecium* nor *S. aureus* isolates. The second gene *lsaE* was found in 69.8% (*n* = 37/53) of Q/d‐resistant *E. faecium* and 35.1% of *E. faecalis* (*n* = 20/57). *Enterococcus faecalis* isolates resistant to Q/D (*n* = 57) carried *ermA* (*n* = 2), *ermB* (*n* = 23), *ermA*/*ermB* (*n* = 5), *ermB*/*vatD* (*n* = 1), *ermB*/*lsaE* (*n* = 15) or *ermA*/*ermB*/*lsaE* (*n* = 5) in addition to *lsaA*, or *lsaA* only (*n* = 6). *Enterococcus faecium* isolates resistant to Q/D (*n* = 53) carried *lsaE* (*n* = 1), *ermB* (*n* = 7), *lsaE*/*ermB* (*n* = 18), *lsaE*/*ermA* (*n* = 1), *ermA*/*ermB* (*n* = 3), *lsaE*/*ermA*/*ermB* (*n* = 16) or *lsaE*/*ermB*/*mefA/E* (*n* = 1). *vgaA*, *vgaB*, *vgaD*, *vatA*, *vatB*, *vatC*, *vatE*, and *vgbB*, are also involved in streptogramins A or B resistance (see Table 1) (Cho et al., 2020; De Graef et al., 2007; Pechère, 2001; Petinaki & Papagiannitsis, 2019; Roberts, 2008) were screened with this array, but not detected. One of the *vat* genes, namely *vatD*, was identified in one *E. faecalis* isolate (VAR‐683) resistant to erythromycin and Q/D. Note, *vatD* is reported in the literature to confer resistance to streptogramins A in *Enterococcus* spp. and being colocated with *ermB* (Hammerum et al., 2001; Jackson et al., 2007; Rende‐Fournier 1993). In contrast with this and according to WGS analysis, *vatD* in VAR‐683 was located on a distinct contig as compared to *ermB*. No resistance gene was detected in 6 Q/d‐resistant enterococcal isolates. In staphylococci, 37 Q/d‐resistant isolates were tested with the array and carried *lsaE* (*n* = 22), *ermA* (*n* = 2), *ermC* (*n* = 2) only or a combination of streptogramins A and/or B resistance genes: *lsaE/ermB* (*n* = 4), *lsaE/ermC* (*n* = 3), *ermB/ermC* (*n* = 1) and *ermA/ermB/ermC* (*n* = 1). *lsaA*, *vga*, *vgb*, and *vat* genes were tested but not detected in staphylococci. Two Q/d‐resistant staphylococcal isolates harbored none of the investigated genes.

Noteworthy, the Q/D resistance mechanisms are still not perfectly understood. Indeed, few studies reported that resistance to streptogramin A is sufficient to confer resistance to Q/D (Hancock, 2005; Yan et al., 2021) while others support that a combination of both resistance to streptogramin A (*vat* or *vga* family) and streptogramin B (*erm* family or *vgbB*) is required to ensure Q/D resistance (Miller et al., 2014; Zarrouk 2000). Both *lsaA* and *lsaE* are the only genes reported to confer resistance to both Q/D components (Alcock et al., 2020; Singh et al., 2002; Wendlandt et al., 2013). In this study, the presence of *lsaA*, *lsaE*, or a combination of both streptogramin A and B resistance genes has been considered concordant with a Q/D resistance phenotype.

### 3.2 Macrolide resistance

Target modification by *erm* genes (coding for 23S rRNA methylases) is known to confer macrolide, lincosamide, and streptogramin B (MLS_B_) resistance and is the most common erythromycin resistance mechanism (Frye & Jackson, 2013; Jensen et al., 1999; Marosevic et al., 2017; Petinaki & Papagiannitsis, 2019; Roberts, 2008; Schwarz et al., 2018; Torres et al., 2018). In our study and as in Frye & Jackson (2013), *ermA* and *ermB* were both detected in enterococci, whereas all three *ermA*, *ermB*, and *ermC* were found in staphylococci. Among the three *ermA*, *ermB*, and *ermC* genes, *ermB* was the most observed in our study, with 92.5% of all erythromycin‐resistant enterococcal isolates carrying this gene (*n* = 98/106) as reported in other studies (Frye & Jackson, 2013; Zaheer et al., 2020). Also, e*rmA* was detected in 34.9% (*n* = 37/106) and found in combination with *ermB* in 30.2% of erythromycin‐resistant isolates (*n* = 32/106). Furthermore, *ermA* was more frequently found in *E. faecium* (46.3% (*n* = 25/54) vs. 23.1% in *E. faecalis* (*n* = 12/52)) whereas *ermB* was identified equally among enterococcal isolates (94.2% *E. faecalis* [*n* = 49/52] and 90.7% *E. faecium* [*n* = 49/54]). Besides, neither *ermC* nor *mphC* was detected in the investigated enterococci isolates. In addition, *mefA/E* involved in macrolide resistance was targeted in this array and found in one *E. faecium* isolate resistant to erythromycin (MIC > 128 mg L^−1^) and Q/D (MIC = 2 mg L^−1^), characterized by the presence of *ermB*, *lsaE*, and *mefA/E*. *mefA/E* was first described in *Streptococcus pneumoniae* to confer macrolide resistance and was also reported in *Enterococcus* spp. from animals and humans (Petsaris et al., 2005). However, the presence of *mefA/E* has been reported to result in low erythromycin MICs (2–16 mg L^−1^) as compared to higher MICs (≥128 mg L^−1^) associated with *erm* genes (Pechère, 2001). In the particular case of a combination of *ermB* and *mefA/E* as observed in this study, inference of *erm* and *mef* genes based on MICs is not possible. No gene was detected in three erythromycin‐resistant enterococcal isolates. In staphylococci, erythromycin resistance was reported in 36/62 isolates and characterized by *ermA* in 5.5% (*n* = 2), *ermB* in 36.1% (*n* = 13), *ermC* in 30.6% (*n* = 11), *ermB/ermC* in 2.8% (*n* = 1), and *ermA/ermB/ermC* in 2.8% (*n* = 1) of resistant isolates. *mefA/E* and *mphC* conferring macrolide resistance were not detected in staphylococci. In eight of these erythromycin‐resistant isolates, no gene was detected.

### 3.3 Lincosamide resistance

While lincosamides (e.g., clindamycin) are not included in the susceptibility testing panel for enterococci, some genes targeted by the array confer resistance to this antimicrobial class: the *lsa*, *vga*, and *erm* genes mentioned above and the *lnuA and lnuB* genes (EFSA; Cho et al., 2020; Lozano et al., 2012). Intrinsic clindamycin resistance is conferred by the presence of *lsaA* in all *E. faecalis* (*n* = 57). Note, *lnuB* was detected in 33.3% and 56.7% of all *E. faecalis* (*n* = 19/57) and *E. faecium* (*n* = 38/67), respectively. In addition, *lnuA*, originally described in staphylococci (Cho et al., 2020; De Graef et al., 2007; Frye & Jackson, 2013) was not detected in enterococci. In staphylococci, several gene combinations were observed among clindamycin‐resistant isolates (*n* = 57) namely, *lsaE* (*n* = 4), *lnuA* (*n* = 1), *lnuB* (*n* = 2), *ermA* (*n* = 2), *ermB* (*n* = 9), *ermC* (*n* = 8), *ermB/ermC* (*n* = 1), *ermA/ermB/ermC* (*n* = 1), *lsaE/ermB* (*n* = 1), *lsaE/lnuB* (*n* = 18), *lsaE/ermB/lnuB* (*n* = 3), and *lsaE/ermC/lnuB* (*n* = 3). One isolate harbored *lnuA* only but did not express clindamycin resistance (MIC = 0.25 mg L^−1^, just below the epidemiological cut‐off value (ECOFF)). Four clindamycin‐resistant isolates did not harbor any relevant gene.

### 3.4 Pleuromutilin resistance

Tiamulin resistance reported in 35 *S. aureus* is known to be conferred by *lsa*, *cfr*, or *vga* genes (Feßler et al., 2018; Schwarz et al., 2018; Van Duijkeren et al., 2014). In this study, 82.9% of tiamulin‐resistant isolates harbored *lsaE* (*n* = 28) alone or in combination with *cfr* (*n* = 1), while *lsaA* and *vga* genes were not detected. Six tiamulin‐resistant isolates did not harbor any relevant gene. AST for tiamulin resistance was not assessed in enterococci.

### 3.5 Tetracycline resistance

Although 59 different tetracycline resistance genes have been described (Marosevic et al., 2017), only the most frequent, *tetM* and *tetO* mediating resistance through ribosomal protection and *tetK* and *tetL* mediating resistance through efflux, were included in this study (Cho et al., 2020; Perreten et al., 2005). In the investigated enterococci isolates, tetracycline resistance was mainly characterized by the presence of *tetM* or a combination of *tetM/tetL* genes. Indeed, *tetM* was identified in 90.5% and 100% of the tetracycline‐resistant *E. faecalis* (*n* = 48/53) and *E. faecium* (*n* = 65/65), respectively. A combination of *tetM/tetL* was reported in 79.2% of *E. faecalis* (*n* = 42/53) and 78.5% of *E. faecium* (*n* = 51/65), as *tetL* was always found in presence of *tetM*. *tetO* was isolated in 9 *E. faecalis* only, either alone (*n* = 5/9), in combination with *tetM* (*n* = 2/9), or with *tetM*/*tetL* (*n* = 2/9). *tetK* was not detected in enterococci in this study. In staphylococci, tetracycline resistance was reported in 98.4% of studied isolates (*n* = 61) and characterized by the presence of at least one *tet* gene, namely *tetK* (*n* = 2) and *tetM* (*n* = 7), or by a combination of them as following *tetM/tetL* (*n* = 12), *tetM/tetK* (*n* = 29), and *tetM/tetL/tetK* (*n* = 11). So, *tetO* was not detected among staphylococci. All tetracycline‐resistant isolates from this study harbored at least one of the four targeted *tet* genes. More specifically, *tetL* was always found in combination with other *tet* genes, with *tetM* (*n* = 91) or *tetM*/*tetO* (*n* = 2) in enterococci, and *tetM* (*n* = 12) or *tetM*/*tetK* (*n* = 11) in staphylococci. In addition, *tetL* and *tetM* were found on the same contig in 19 of 25 (76.0%) sequenced enterococci, adjacent in 18 of them. In the latter, *tetL* and *tetM* were separated by ~3 kb but no insertion sequence (IS) was found between the two genes.

### 3.6 Oxazolidinone resistance

In this study, all linezolid‐resistant (LZD‐R) isolates (*n* = 22) collected through the Belgian official 2019–2020 monitoring of enterococci in food‐producing animals were assayed. The *cfr*, *optrA*, and *poxtA* genes were targeted by this array. All linezolid‐resistant isolates were harboring at least one linezolid‐resistance gene: *optrA* (*n* = 14), *poxtA* (*n* = 2), or *optrA/poxtA* (*n* = 6). However, *cfr* was not detected among enterococci of this study, yet found in enterococci in Timmermans et al. (2022a). Linezolid resistance was not observed among the studied staphylococci isolates, and neither were *optrA* and *poxtA* detected. However, one linezolid‐susceptible and chloramphenicol‐resistant isolate characterized with an LZD MIC of 4 mg L^−1^ was harboring *cfr*, which presence was confirmed by WGS. Additionally, the presence of *optrA* and *poxtA* is not restricted to linezolid‐resistant isolates since 9/15 enterococcal isolates exhibiting the LZD MIC of 4 mg L^−1^ (considered susceptible according to the ECOFF), were also harboring *optrA* (*n* = 6), *poxtA* (*n* = 2) or both (*n* = 1). Eight out of these nine isolates were resistant to chloramphenicol (MIC > 32 mg L^−1^) and one was susceptible to chloramphenicol (MIC = 32 mg L^−1^) as well. These genes were also found in enterococcal isolates characterized by a MIC of 2 mg L^−1^ (*n* = 5/60, 4 *optrA*, and 1 *poxtA*) and 1 mg L^−1^ (*n* = 2/27, 1 *optrA*, and 1 *poxtA*). Among these seven isolates, four were resistant to chloramphenicol (MIC > 32 mg.L^−1^) and three were susceptible to chloramphenicol (MIC = 16 mg L^−1^ [*n* = 2] and MIC = 32 mg L^−1^ [*n* = 1]).

### 3.7 Phenicol resistance

Among chloramphenicol‐resistant‐enterococci, genes coding for *cat*_pC194_ (*n* = 2), *cat*_pC221‐223_ (*n* = 4), *optrA* (*n* = 1), *poxtA* (*n* = 2), *optrA*/*cat*_pC221‐223_ (*n* = 1), *fexA*/*optrA* (*n* = 19), *optrA*/*poxtA* (*n* = 3), and *fexA*/*optrA*/*poxtA* (*n* = 3) were detected. Surprisingly, *cat*_pC194_ was detected in seven isolates phenotypically susceptible to chloramphenicol with MICs ranging from 16 mg L^−1^ (*n* = 1) to 32 mg L^−1^ (*n* = 6). Four enterococci harboring a *fexA/optrA* combination were characterized by a chloramphenicol MIC of 16 mg L^−1^ (*n* = 1) or 32 mg L^−1^ (*n* = 3). Similarly, *optrA*, *poxtA*, or a combination of both was found in 1, 4, and 1 chloramphenicol‐susceptible isolates, respectively, characterized by MICs between 16 mg L^−1^ (*n* = 2) and 32 mg L^−1^ (*n* = 4). Among the 12 chloramphenicol‐resistant staphylococci, nine carried the *fexA* gene, one carried *cfr*, while no relevant genes were detected in the two remaining isolates. Neither *cat* nor *optrA* or *poxtA* was detected in these chloramphenicol‐resistant staphylococci. Three chloramphenicol‐susceptible isolates harbored *fexA* and were characterized by a MIC of 8 mg L^−1^ (*n* = 2) or 16 mg L^−1^ (*n* = 1) for chloramphenicol. Besides the direct cross‐resistance to linezolid and phenicols conferred by *cfr*, *optrA*, and *poxtA*, the concomitant presence of *optrA* and/or *poxtA* with the phenicol resistance gene *fexA* was observed in this study in 81.3% (*n* = 26/32) of the enterococcal isolates, as reported elsewhere (Brenciani et al., 2018; Ruiz‐Ripa et al., 2020; Sadowy, 2018; Timmermans et al., 2022a). In addition, *fexB* was not included in the array but was detected in 10 sequenced enterococcal isolates, including five chloramphenicol‐susceptible strains characterized by a MIC of 32 mg L^−1^. *fexB* was detected together with *poxtA* (*n* = 3), *optrA/poxtA* (*n* = 5), or *fexA/optrA/poxtA* (*n* = 2). Genome analysis indicated that the *optrA/fexA* combination was found on the same contig in 87.5% of isolates (*n* = 15/16). *optrA* and *fexA* were close to each other (~700 bp) in 14 of them and more distant in the remaining isolate (~5 kb). No IS was found between *optrA* and *fexA* in any of these isolates. The *poxtA/fexB* combination was never associated with the same contig, as described elsewhere (Freitas et al., 2020; Ruiz‐Ripa et al., 2020; Timmermans et al., 2022a).

### 3.8 Glycopeptide resistance

Although rarely observed in 2019 and 2020 through the Belgian AMR monitoring program in enterococci, vancomycin resistance was however another point of interest of this study. Four probes targeting *vanA*, *vanB*, *vanC*_*1*,_ and *vanC*_*2‐3*_ genes were included in this array. *vanA* was detected in one of the two vancomycin‐resistant *E. faecalis* isolates whereas *vanB* was not detected. Sequencing of the second isolate (VAR‐660) revealed the unexpected presence of *vanL*. As expected, *vanC*_*1*_ and *vanC*_*2‐3*_, intrinsic to *E. gallinarum* and *E. casseliflavus*, respectively, were not detected. Vancomycin resistance being absent in staphylococci from the studied years, no vancomycin‐resistant isolates were tested with this array and in line with this, no *van* genes were detected with the array.

### 3.9 Aminoglycoside resistance

Many aminoglycoside resistance genes were described in the literature (Frye & Jackson, 2013; Hollenbeck & Rice, 2012; Schwarz et al., 2018; Strauss et al., 2015). The most common were selected and tested with this array, namely *aadD*, *aadE*, *aacA‐aphD*, *aphA3*, and *aph2‐Id‐Ie*. In enterococci, gentamicin resistance was characterized by the presence of *aacA‐aphD* in all resistant isolates (*n* = 37). Note, *aph2‐Id‐Ie* was not detected. Other genes conferring resistance to aminoglycosides not in the scope of the susceptibility testing (kanamycin and streptomycin) were also detected in enterococci, with *aadD*, *aphA3*, and *aadE* found in 2.4% (*n* = 3/124), 47.6% (*n* = 59/124), and 60.5% (*n* = 75/124) of all isolates, respectively. In staphylococci, susceptibility to gentamicin, kanamycin, and streptomycin was assessed by BMD testing. Kanamycin‐resistant isolates (*n* = 19) were characterized by the presence of *aacA‐aphD/aadD* (*n* = 12/19, 63.2%), *aacA‐aphD* alone (*n* = 5/19, 26.3%) or *aadD* alone (*n* = 1/19, 5.3%). No aminoglycoside resistance gene could be detected in one kanamycin‐resistant isolate. On the other hand, *aadD* was identified in seven kanamycin‐susceptible isolates. Resistance to kanamycin was commonly associated with gentamicin resistance (*n* = 18/19) in accordance with the finding of gene combinations such as *aacA‐aphD* (*n* = 5) or *aacA‐aphD/aadD* (*n* = 12) in gentamicin‐resistant isolates (*n* = 19). No aminoglycoside resistance gene targeted by the array could be detected in two gentamicin‐resistant isolates. Resistance to streptomycin (*n* = 11) was correlated with the presence of *aadE* in four isolates. No aminoglycoside resistance gene could be detected with the array in the seven remaining streptomycin‐resistant isolates. Neither *aphA3* nor *aph2‐Id‐Ie* could be detected in any of the investigated aminoglycoside‐resistant isolates.

### 3.10 Diaminopyrimidine resistance

Although susceptibility to trimethoprim was not assessed by BMD assays, *dfr* resistance genes were screened in enterococci. Among the 4 tested *dfr* genes, *dfrD*, *dfrK*, and *dfrG* were found in 1, 2, and 61 isolates, respectively. More precisely, *dfrD* was detected in 1 *E. faecalis*, *dfrK* in 2 *E. faecium*, and *dfrG* was detected in 35 *E. faecalis* and 26 *E. faecium* isolates. Also, *dfrA/C* was not detected in enterococci. Among the 55 trimethoprim‐resistant staphylococcal isolates, the most previously reported *dfr* genes (Schwarz et al., 2018; Strauss et al., 2015; Wendlandt et al., 2013), namely *dfrA/C*, *dfrD*, *dfrG*, and *dfrK*, were targeted and found in 1, 0, 25 and 22 isolates, respectively.

### 3.11 Comparison of array‐genotype with WGS‐genotype: Short investigation

The AMR genetic profile of 31 isolates (16 *E. faecalis*, 14 *E. faecium*, and 1 MRSA) was investigated by whole‐genome sequencing and compared to data resulting from the array. The selection of isolates for WGS was based on resistance to linezolid (*n* = 22), vancomycin (*n* = 2), or a minimum of 5 antimicrobials (*n* = 7). The genetic profiles gathered with the AMR array were all confirmed by WGS analyses (31/31) (see Table 5). These results ensured the reliability of the array method. Conversely, WGS investigation highlighted genes not covered by this array, that is *vanL* found in one vancomycin‐resistant isolate (VAR‐660).

**Table 5:** Comparison of the AMR genes detected by the array with WGS results

**Isolate ID**	**Biosample ID**	**Species**	**Phenotypic resistance profile**	**Genetic resistance profile detected by the array and/or by WGS**	**Concordance ARRAY versus WGS (%)**
VAR‐660	SAMN30360174	*Enterococcus faecalis*	SYN VAN	*lsaA*, *vanL* 2	100
VAR‐662	SAMN30360175	*Enterococcus faecalis*	CHL ERY GEN LZD SYN TET	*aacA‐aphD*, *aadE*, *aphA3*, *dfrG*, *ermB*, *fexA*, *lsaA*, *lsaE*, *lnuB*, *optrA*, *tetL*, *tetM*	100
VAR‐663	SAMN30360176	*Enterococcus faecalis*	CHL ERY GEN LZD SYN TET	*aacA‐aphD*, *aphA3*, *dfrG*, *ermA*, *ermB*, *fexA*, *lsaA*, *optrA*, *tetL*, *tetM*	100
VAR‐665	SAMN30360177	*Enterococcus faecalis*	CHL ERY LZD SYN	*ermA*, *fexA*, *lsaA*, *optrA*	100
VAR‐668	SAMN30360178	*Enterococcus faecalis*	CHL CIP ERY GEN LZD SYN TET	*aacA‐aphD*, *aadE*, *aphA3*, *dfrG*, *ermB*, *fexA*, *lsaA*, *lsaE*, *lnuB*, *optrA*, *tetL*, *tetM*	100
VAR‐673	SAMN30360179	*Enterococcus faecalis*	CHL CIP ERY GEN SYN TET	*aacA‐aphD*, *aadE*, *aphA3*, *dfrG*, *ermB*, *fexA*, *lsaA*, *optrA*, *tetL*, *tetM*	100
VAR‐675	SAMN30360180	*Enterococcus faecalis*	CHL ERY LZD SYN TET	*ermA*, *ermB*, *fexA*, *lsaA*, *optrA*, *tetL*, *tetM*	100
VAR‐676	SAMN30360181	*Enterococcus faecalis*	CHL ERY GEN LZD SYN TET	*aacA‐aphD*, *aadE*, *aphA3*, *ermA*, *ermB*, *fexA*, *lsaA*, *lsaE*, *lnuB*, *optrA*, *tetM*	100
VAR‐677	SAMN30360182	*Enterococcus faecalis*	CIP LZD SYN TET	*dfrG*, *fexA*, *lsaA*, *optrA*, *tetL*, *tetM*	100
VAR‐678	SAMN30360183	*Enterococcus faecalis*	CHL ERY LZD SYN TET	*dfrG*, *ermA*, *fexA*, *lsaA*, *optrA*, *tetL*, *tetM*	100
VAR‐682	SAMN30360184	*Enterococcus faecalis*	CHL ERY LZD SYN TET	*ermB*, *fexA*, *lsaA*, *optrA*, *tetL*, *tetM*	100
VAR‐683	SAMN30360185	*Enterococcus faecalis*	ERY SYN TET VAN	*aadD*, *aadE*, *aphA3*, *dfrD*, *lsaA*, *ermB*, *tetL*, *tetM*, *vanA*, *vatD*	100
VAR‐685	SAMN30360186	*Enterococcus faecalis*	SYN TET	*lsaA*, *tetL*, *tetM*	100
VAR‐686	SAMN30360187	*Enterococcus faecalis*	CHL CIP ERY GEN SYN TET	*aacA‐aphD*, *aadE*, *aphA3*, *dfrG*, *ermA*, *ermB*, *fexA*, *lsaA*, *lsaE*, *lnuB*, *optrA*, *tetL*, *tetM*	100
VAR‐688	SAMN30360188	*Enterococcus faecalis*	ERY LZD SYN TET	*aadE*, *aphA3*, *ermA*, *ermB*, *fexA*, *lsaA*, *optrA*, *tetL*, *tetM*	100
VAR‐689	SAMN30360189	*Enterococcus faecalis*	CHL ERY GEN LZD SYN TET	*aacA‐aphD*, *aadE*, *aphA3*, *dfrG*, *ermA*, *ermB*, *fexA*, *lsaA*, *lsaE*, *lnuB*, *optrA*, *tetL*, *tetM*	100
VAR‐661	SAMN30360190	*Enterococcus faecium*	AMP DAP ERY GEN SYN TET	*aacA‐aphD*, *aadE*, *aphA3*, *dfrG*, *ermA*, *ermB*, *lsaE*, *lnuB*, *tetL*, *tetM*	100
VAR‐664	SAMN30360191	*Enterococcus faecium*	AMP ERY GEN SYN TET	*aacA‐aphD* 1, *aadE*, *aphA3*, *dfrG*, *ermB*, *lsaE*, *lnuB*, *poxtA*, *tetL*, *tetM*	100
VAR‐666	SAMN30360192	*Enterococcus faecium*	CHL ERY LZD TET	*ermA*, *fexA*, *optrA*, *poxtA*, *tetL*, *tetM*	100
VAR‐667	SAMN30360193	*Enterococcus faecium*	CHL ERY GEN LZD SYN TET	*aacA‐aphD*, *aadE*, *aphA3*, *dfrG*, *ermA*, *ermB*, *lsaE*, *lnuB*, *optrA*, *poxtA*, *tetL*, *tetM*	100
VAR‐669	SAMN30360194	*Enterococcus faecium*	CHL LZD SYN TET	*aadE*, *ermA*, *fexA*, *lsaE*, *lnuB*, *optrA*, *tetL*, *tetM*	100
VAR‐670	SAMN30360195	*Enterococcus faecium*	ERY GEN LZD SYN TET	*aacA‐aphD*, *aadE*, *aphA3*, *dfrG*, *ermA*, *ermB*, *lsaE*, *lnuB*, *optrA*, *poxtA*, *tetL*, *tetM*	100
VAR‐671	SAMN30360196	*Enterococcus faecium*	ERY LZD TET	*dfrG*, *ermA*, *ermB*, *optrA*, *tetL*, *tetM*	100
VAR‐672	SAMN30360197	*Enterococcus faecium*	CHL ERY LZD TET	*dfrG*, *ermA*, *optrA*, *poxtA*, *tetM*	100
VAR‐674	SAMN30360198	*Enterococcus faecium*	AMP DAP ERY GEN SYN TET	*aacA‐aphD*, *aphA3*, *aadE*, *cat*p_C194_, *ermA*, *ermB*, *lsaE*, *lnuB*, *tetL*, *tetM*	100
VAR‐679	SAMN30360199	*Enterococcus faecium*	CHL ERY LZD SYN TET	*aadE* 1, *aphA3*, *dfrG*, *ermB*, *lsaE*, *lnuB*, *optrA*, *poxtA*, *tetL*, *tetM*	100
VAR‐680	SAMN30360200	*Enterococcus faecium*	LZD TET	*aadD*, *poxtA*, *tetL*, *tetM*	100
VAR‐681	SAMN30360201	*Enterococcus faecium*	LZD SYN TET	*poxtA*, *tetL*, *tetM*	100
VAR‐684	SAMN30360202	*Enterococcus faecium*	CHL LZD TET	*fexA*, *optrA*, *poxtA*, *tetM*	100
VAR‐687	SAMN30360203	*Enterococcus faecium*	ERY LZD TET	*dfrG*, *ermA*, *ermB*, *optrA*, *poxtA*, *tetL*, *tetM*	100
VAR 659	SAMN30360173	*Staphylococcus aureus*	CHL CIP CLI ERY FOX PEN STR SYN TET TIA TMP	*aadD*, *aadE*, *cfr*, *dfrG*, *ermB*, *lsaE*, *lnuB*, *tetK*, *tetL*, *tetM*	100

*Note*: Genetic profiles include targets of this array and highlight consistency in comparison to WGS. Each gene detected by WGS was compared to the reference gene with a minimum percentage of coverage of 60% and a minimum percentage of identity of 90%.

^1^ Gene detected by WGS compared to the reference gene with a minimum percentage of coverage of 40% and a minimum percentage of identity of 40%.

^2^ Gene detected by WGS, not targeted by the ARRAY.

## 4 DISCUSSION

Among the entire collection of 62 staphylococci and 124 enterococci investigated, a total of 91.3% (*n* = 718/786) resistance profiles demonstrated experimentally could be associated with the AMR gene targeted by the array, namely 89.2% (*n* = 305/342) in staphylococci and 93.0% (*n* = 413/444) in enterococci. This result supports that the most common resistance genes were targeted by our array since a genetic marker could be associated with more than 90.0% of the resistant phenotypes leaving only a few without genetic information.

This study highlighted the coexistence of both *lsaA* and *lsaE* in 20/57 (35.1%) of the *E. faecalis* isolates, suggesting the presence of a selection pressure since *lsa* genes are involved in other phenotypic resistances such as pleuromutilin (tiamulin) and lincosamide (clindamycin) resistance as reported in other studies (Feßler et al., 2018; Hollenbeck & Rice, 2012). However, antimicrobial susceptibility for these antimicrobial classes is not monitored in enterococci in official surveillance programs. In addition, *lsaE* was the most observed gene in MRSA with 29 isolates showing resistance to Q/D (*n* = 29/37; 78.4%), as well as to tiamulin (*n* = 29/35; 88.6%) and clindamycin (*n* = 29/57; 50.9%). Such pleuromutilin‐lincosamide‐streptogramin A resistance mediated by *lsaE* as found in our study among *Staphylococcus* spp. and *Enterococcus* spp. isolated from animals was reported elsewhere in animals and also in humans (Feßler et al., 2018; Schwarz et al., 2018; Wendlandt et al., 2013).

Overall, *ermB* was the most prevalent macrolide resistance gene in our collection (in 79.6% of all erythromycin‐resistant isolates, *n* = 113/142) and particularly among enterococci (*n* = 98/106 vs. *n* = 15/36 staphylococci), as described in Frye & Jackson (2013). The *erm* genes were more frequently observed in enterococci than staphylococci (97.2% vs. 77.8%) as first described in Jensen et al. (1999). Interestingly, other studies (Jensen et al., 1999; Petinaki & Papagiannitsis, 2019) also reported *ermC* mostly in staphylococci and in rare cases in enterococci.

*lnuB* was the second most observed lincosamide resistance gene in staphylococci as found in 45.6% of isolates (*n* = 26), however, it has been mostly described in enterococci (Cho et al., 2020; Feßler et al., 2018) and rare cases in staphylococci (Li et al., 2013; Lozano et al., 2012; Wendlandt et al., 2013).

All tetracycline‐resistant isolates of this study harbored at least one of the tested genes, namely *tetL*, *tetM*, *tetK*, and/or *tetO*, with *tetM* being the most prevalent one as reported elsewhere (Argudin et al., 2017; Cho et al., 2020; Frye & Jackson, 2013; Schwarz et al., 2018). So, *tetL* was always found in combination with other *tet* genes, particularly with *tetM* which was adjacent in 72.0% (*n* = 18/25) of the isolates investigated by WGS. This result suggests that *tetM* and *tetL* might be transferable together thereby spreading two different mechanisms of resistance, ribosomal protection, and proteins efflux, respectively.

In this study, at least one linezolid‐resistance gene was detected in each LZD‐R strain: *optrA*, *poxtA*, or a combination of both genes in 63.6%, 9.1%, and 27.3% of LZD‐R enterococcal isolates, respectively. These genes were also found in LZD‐susceptible isolates characterized by a MIC of 4 mg L^‐1^ (*n* = 9/15), 2 mg L^‐1^ (*n* = 5/60), or 1 mg L^‐1^ (*n* = 2/27). These results support a recent warning from EFSA suggesting that all strains displaying MICs ≥ 4 mg L^‐1^ for linezolid and exhibiting resistance to the other compounds typically conferred by *cfr*, should be screened for this gene (European Food Safety Authority & European Centre for Disease Prevention and Control, 2022, p. 116). Perhaps it should be considered to extend it to *optrA* and *poxtA* screening as well, especially as they have been mostly found in enterococci (Timmermans et al., 2022a). In line with the observation of *optrA* or *poxtA* in LZD‐susceptible isolates, previous studies (Dejoies et al., 2020; Timmermans et al., 2022a) demonstrated that a 48 h‐incubation conducted during susceptibility testing enhanced the detection of isolates carrying linezolid‐resistance determinants suggesting that *optrA* and *poxtA* might be inducible. However, more experiments are required at this point. Few chloramphenicol susceptible isolates were also characterized by the presence of a resistance gene such as *fexA* (*n* = 4) and *cat*_pC194_ (*n* = 7) as described for LZD‐susceptible isolates here above. *cat*_pC194_, *cat*_pC221_, and *cat*_pC223_ from the *cat* enzyme family as well as *fex* exporters are inducible (Schwarz et al., 2016), which could explain the variations observed among MICs. This highlights the limitations of AST based on phenotypic cut‐offs for the screening of AMR genes, in particular for such inducible genes. In addition, future genetic investigation of chloramphenicol phenotypic resistance may partially rely on the detection of *fexB*, a member of the *fex* exporters family already reported in enterococci (Argudin et al., 2017; Schwarz et al., 2018) and found in 10 sequenced enterococci of this study. Interestingly, the concomitant presence of *optrA* and *fexA* was observed in 23 of our isolates independently of phenotypic resistance profiles. Particularly, *optrA* was found close to *fexA* in 14 of the 16 isolates investigated by WGS. The absence of IS between the two genes in these isolates suggests both genes might be transferable together.

Vancomycin resistance, although rare, was characterized by the presence of the *vanA* cluster in one of the two isolates, the most common gene reported in other studies (Courvalin, 2006; Ekwanzala et al., 2020; Torres et al., 2018). In the remaining vancomycin‐resistant isolate (VAR‐660), sequencing highlighted the presence of the rare *vanL*, a gene not covered by this array. The rare *vanL* gene cluster, first described by Boyd et al. (2008), has been so far detected on the chromosome of a single *E. faecalis* isolate of human origin (Ekwanzala et al., 2020) displaying low‐level vancomycin resistance (Ekwanzala et al., 2020; Boyd et al., 2008) as observed here (MIC = 8 mg L^−1^). Our study reports the presence of *vanL* in an *E. faecalis* isolate from animal origin. The origin of the *vanL* gene cluster and the way an animal (i.e., a pig) acquired this strain remain elusive.

Gentamicin resistance was characterized by the presence of *aacA‐aphD* in all resistant enterococci (*n* = 37) and by *aacA‐aphD* (*n* = 5) or *aacA‐aphD/aadD* (*n* = 12) in resistant staphylococci (*n* = 19) of this study. In addition, resistance to gentamicin was found to be associated with kanamycin resistance in 18 of these 19 resistant staphylococci, with kanamycin‐resistant isolates (*n* = 19) harboring *aadD* alone (*n* = 1), *aacA‐aphD* (*n* = 5) or *aacA‐aphD/aadD* (*n* = 12) gene combination.

In a few cases of this study (10.8% of the individual resistant phenotypes in staphylococci and 7.0% in enterococci), a genetic marker could not be associated with the resistant phenotype. In enterococci, no relevant AMR genes were found with the array to explain erythromycin (*n* = 3), Q/D (*n* = 6), chloramphenicol (*n* = 10), gentamicin (*n* = 1) and vancomycin (*n* = 1) phenotypic resistances (out of a total of 444 assessed phenotypes). In staphylococci, a genetic explanation was missing for clindamycin (*n* = 1), erythromycin (*n* = 6), Q/D (*n* = 2), tiamulin (*n* = 6), chloramphenicol (*n* = 2), gentamicin (*n* = 2), streptomycin (*n* = 7), and trimethoprim (*n* = 7) resistant phenotypes (out of a total of 342 assessed phenotypes). In addition, the results of this study highlighted the complexity of Q/D resistance and a lack of a complete genetic explanation in 14/90 isolates since the identified genes (except for *lsaA* and *lsaE*) have been reported to confer resistance to either streptogramin A or streptogramin B but not both. Particularly, the presence of *erm* (streptogramin B) alone in Q/d‐resistant isolates (10/53 *E. faecium*, 4/37 MRSA) does not explain the observed Q/d‐resistant phenotype. Similar to *lsa*, *eatA* would be an interesting target as this gene confers the same profile of cross‐resistance to lincosamides, streptogramins A, and pleuromutilins (Isnard et al., 2013). The WGS investigation revealed the presence of *msrC* in one *E. faecium* (VAR‐681) exhibiting resistance to Q/D, this gene being frequently reported elsewhere in this species (Frye & Jackson, 2013; Hollenbeck & Rice, 2012). And, *msrC* has been described in Q/d‐susceptible isolates, however first reported as being specifically found in resistant *E. faecium*. This suggests that it might be silent or involved in resistance to other antibiotics (Smoglica et al., 2022) such as macrolides (Portillo et al., 2000; Zaheer et al., 2020). Therefore, *msrA* which shares significant sequence identity with *msrC* has been reported to confer macrolides‐streptogramin B resistance in staphylococci and could be an interesting future target to investigate erythromycin and/or Q/D phenotypic resistances (Reynolds & Cove, 2005). In addition, *ermF*, *ermY*, *mphB* or more recently described *mefD*, *msrF*, and *msrH* have been found in staphylococci to confer macrolide and lincosamide resistances (Miklasinska‐Majdanik, 2021; Schwarz et al., 2018; Woodford, 2005). Besides, *ereA* and *ereB* esterases have been reported to confer macrolide resistance in staphylococci isolated from animals as well (Miklasinska‐Majdanik, 2021; Schwarz et al., 2018). In addition, chromosomal mutations in *rplD* or *rplV* coding for ribosomal proteins L4 and L22 leading macrolide and Q/D resistance, respectively, were also observed in *Streptococcus pneumoniae* and *S. aureus* and could be targeted in future studies (Farrell et al., 2004; Malbruny et al., 2002; Miklasinska‐Majdanik, 2021). The *sal* gene family reported to confer lincosamide, streptogramin A and pleuromutilin resistance in staphylococci from humans or animals at various levels could be investigated in an attempt to explain the tiamulin‐resistant phenotypes observed in 6/35 isolates (Mohamad et al., 2022; Schwarz et al., 2018). Pleuromutilin resistance also derives from chromosomal mutations in 23S rRNA or *rplC* as described in staphylococci isolated from both humans and animals (Paukner & Riedl, 2017; Van Duijkeren et al., 2014). No gene of this array was detected in 10 isolates (1 *E. faecalis* and 9 MRSA) exhibiting resistance to aminoglycosides, namely to gentamicin (*n* = 2 MRSA and 1 *E. faecalis*) and to streptomycin (*n* = 7 MRSA). In enterococci, an intrinsic low‐level of aminoglycoside resistance can be occasionally observed due to the presence of the species‐specific chromosomal *aac(6')‐Ii* gene found in almost all *E. faecium* (Adamecz et al., 2021). Among many aminoglycosides modifying enzymes (AMEs) described in the literature, a few of them such as *str* could be interesting to target in the future as already described in staphylococci (Ramirez & Tolmasky, 2010; Schwarz et al., 2018). In contrast, few cases of mismatch were observed with kanamycin‐susceptible isolates (*n* = 7 MRSA) by BMD testing carrying one gene encoding aminoglycosides resistance (*aadD* in this study), as already reported in other studies (Adamecz et al., 2021; Feizabadi et al., 2006; Yean et al., 2007). This could be explained if this gene is nonfunctional and could be investigated in future studies. In addition, Ida et al. (2002) have shown that rearrangements caused by the integration of insertion elements into the staphylococcal chromosome or plasmids affected the expression of adjacent genes including aminoglycoside resistance genes. Finally, 7/55 (12.7%) trimethoprim‐resistant MRSA isolates were not found to be associated with the presence of a genetic marker in this study. The presence of other members of the *dfr* genes family (e.g., *dfrF*) or the presence of chromosomal mutations may explain the phenotypic resistance observed in these isolates (Woodford, 2005).

## 5 CONCLUSIONS

In this study, a bead array was described aiming to detect broad AMR genetic profiles of Gram‐positive bacteria from healthy animals in a single experiment. A number of resistance genes circulating among *Enterococcus* spp. and *Staphylococcus* spp. were targeted by this array allowing the screening of a large number of strains in a limited time and at an affordable cost. The relatively short turnaround time (~8 h) and the use of a software source code freely available allow rapid data acquisition and analysis. In addition, the method is cost‐effective with a reagent price of 18 €/sample. The designed array targeted AMR from 9 antimicrobial families, including resistance to critically important antimicrobials, linezolid, and vancomycin, which are important to monitor in both human and animal sectors. Due to the flexibility of the method, a more specific bead array (e.g., linezolid‐array) could also be easily designed by removing/adding probes to respond to a particular epidemiologic situation (i.e., location) or to screen other bacterial species; and could also be extended to other research areas (i.e., virulence). Animal isolates were investigated; nevertheless, other reservoirs of AMR genes (humans, environment) could be assessed with the array. Besides AMR genes, resistance resulting from point mutations in receptors (e.g., ciprofloxacin resistance) or in specific proteins (e.g., daptomycin resistance) are targetable as well. In parallel to the official monitoring based on AST, screening with the array allowed us to rapidly determine genetic profiles circulating among livestock animals in Belgium. Isolates of this study have frequently been shown to carry two or more resistance genes conferring the same resistance phenotype, and sometimes genes from the same family, for example *erm* or *tet* genes. This accumulation of genes has been frequently observed while a single gene is sufficient to confer resistance, and may be explained by acquisition at different times and/or under different conditions, including their possible co‐location on plasmids carrying multiple resistance genes even in absence of selective pressure (Schwarz et al., 2018). Many studies suggested that commensal bacteria are probable reservoirs for AMR genes and can transfer these to pathogenic organisms, for example *Bacillus* spp. or *Salmonella* spp. (Frye & Jackson, 2013; Schwarz et al., 2018). Also, while the spread of resistance genes such as *ermB* and *tetM* is limited to Gram‐positive bacteria, genes such as *aadE* or *tetL* are found both in Gram‐positive such as reported here but also in Gram‐negative bacteria (*E. coli*) as reported elsewhere (Frye & Jackson, 2013; Schwarz et al., 2018). This array allowed the identification of AMR genes in resistant isolates, spotting a few isolates without genetic explanation as interesting candidates for WGS to identify new resistance mechanisms. This approach allowed us to demonstrate the presence of *vanL* in one *E. faecalis* isolate from animal origin. Our study also highlighted the presence of the *lnuB* gene in staphylococci, rarely reported in the literature. Finally, the missing concordance between aminoglycoside resistance genes and phenotypic resistance observed in this study illustrates the importance of still relying on the routine phenotypic susceptibility test for resistance monitoring. Oppositely, the presence of an oxazolidinone resistance gene in a susceptible isolate occurred several times and showed the importance of genotyping as a complement to phenotyping, particularly for such potentially inducible genes. Our data stress the importance of interpreting AMR with caution and the complementarity of both phenotyping and genotyping methods.

## AUTHOR CONTRIBUTIONS

**Carole Kowalewicz**: Conceptualization (lead); formal analysis (lead); methodology (lead); writing – original draft (lead); writing – review and editing (equal). **Michael Timmermans**: Conceptualization (supporting); methodology (supporting); writing – review and editing (supporting). **David Fretin**: Writing – review and editing (supporting). **Pierre Wattiau**: Conceptualization (supporting); supervision (supporting); writing – review and editing (supporting). **Cécile Boland**: Conceptualization (supporting); formal analysis (supporting); methodology (supporting); supervision (lead); writing – original draft (supporting); writing – review and editing (equal).

## CONFLICT OF INTEREST

None declared.

## ETHICS STATEMENT

None required.

## Data Availability

All data analyzed during this study are included in this published article. The WGS datasets are available under BioProject PRJNA868571: https://www.ncbi.nlm.nih.gov/bioproject/PRJNA868571.

## Appendix

**Table A1:** List of control strains used for the array validation and their source

**Species**	**Strain ID**	**Gene**	**Source**
*Enterococcus faecalis*	VAR‐473	*cfr*	Timmermans et al. (2022a)
*Enterococcus faecalis*	VAR‐473	*poxtA*	Timmermans et al. (2022a)
*Enterococcus faecalis*	VAR‐473	*optrA*	Timmermans et al. (2022a)
*Enterococcus faecium*	VAR‐182	*fexA*	In‐house collection
*Enterococcus* spp.	VAR‐181	*vanA*	In‐house collection
*Enterococcus faecalis*	V583	*vanB*	EURL‐AR
*Enterococcus gallinarum*	VAR‐530	*vanC*_*1*_	Timmermans et al. (2022a)
*Enterococcus casseliflavus*	VAR‐484	*vanC*_*2‐3*_	Timmermans et al. (2022a)
*Enterococcus faecium*	VAR‐172	*cat*p _C194_	In‐house collection
*Enterococcus* spp.	VAR‐181	*cat*p _C221‐223_	In‐house collection
*Enterococcus casseliflavus*	UC 73 Id	*aph2‐Id/Ie*	EURL‐AR
*Staphylococcus aureus*	VAR‐141	*aacA‐aphD*	In‐house collection
*Enterococcus faecium*	VAR‐182	*aphA3*	In‐house collection
*Staphylococcus aureus*	VAR‐141	*aadD*	In‐house collection
*Enterococcus faecium*	VAR‐182	*aadE*	In‐house collection
*Enterococcus faecium*	S. A plasmid pG01	*dfrA/C*	Caryl & O'Neill (2009)
*Enterococcus* spp.	VAR‐181	*dfrD*	In‐house collection
*Enterococcus faecium*	VAR‐182	*dfrG*	In‐house collection
*Snterococcus aureus*	VAR‐134	*dfrK*	In‐house collection
*Streptococcus* spp.	01D19	*mefA*	Internal Sciensano collection
*Streptococcus* spp.	02J1175	*mefE*	Internal Sciensano collection
*Enterococcus faecium*	VAR‐182	*ermA*	In‐house collection
*Enterococcus faecium*	VAR‐182	*ermB*	In‐house collection
*Bacillus subtilis*	B.3HU104/pE194	*ermC*	EURL‐AR
*Staphylococcus chromogenes*	TS1 ‐ NC_007768.1	*lnuA*	Luthje et al. (2007)
*Enterococcus faecium*	VAR‐182	*lnuB*	In‐house collection
*Enterococcus faecalis*	VAR‐175	*lsaA*	In‐house collection
*Enterococcus faecium*	VAR‐182	*lsaE*	In‐house collection
*Staphylococcus aureus*	BM 3093, pIP680	*vatA*	EURL‐AR
*Staphylococcus* spp.	9674438‐1vatB‐pos.	*vatB*	EURL‐AR
*Staphylococcus cohnni*	BM10711, pIP1675	*vatC*	EURL‐AR
*Enterococcus faecium*	BM4145	*vatD*	EURL‐AR
*Enterococcus faecium*	UW1965	*vatE*	EURL‐AR
*Staphylococcus aureus*	VAR‐134	*vgaA*	In‐house collection
*Staphylococcus aureus*	BM12235, pIP1633	*vgaB*	EURL‐AR
*Staphylococcus cohnni*	BM10711, pIP1675	*vgbB*	EURL‐AR
*Staphylococcus xylosus*	LT223129.1 (JW2311)	*mphC*	Wipf et al. (2017)
*Enterococcus faecium*	VAR‐182	*tetM*	In‐house collection
*Enterococcus faecium*	VAR‐182	*tetL*	In‐house collection
*Staphylococcus aureus*	VAR‐143	*tetK*	In‐house collection
*Enterococcus faecalis*	VAR‐175	*tetO*	In‐house collection
*Staphylococcus aureus*	ATCC 29213	*staph*	ATCC collection
*Enterococcus faecalis*	ATCC 29212	*sodA‐fs*	ATCC collection
*Enterococcus faecium*	*E. faecium* 2013/16227	*sodA‐fm*	In‐house collection

*Note*: Positive control of vgaD was absent in this study.

Strains with source as “In‐house collection” were isolates sequenced by whole‐genome sequencing of our collection.
